# Regulating the immunosuppressive tumor microenvironment to enhance breast cancer immunotherapy using pH-responsive hybrid membrane-coated nanoparticles

**DOI:** 10.1186/s12951-021-00805-8

**Published:** 2021-02-25

**Authors:** Chunai Gong, Xiaoyan Yu, Wei Zhang, Lu Han, Rong Wang, Yujie Wang, Shen Gao, Yongfang Yuan

**Affiliations:** 1 Department of Pharmacy, Shanghai Ninth People’s Hospital, Shanghai JiaoTong University School of Medicine, Shanghai, 201999 China; 2 Department of Pharmaceutics, Shanghai Pulmonary Hospital, Tongji University School of Medicine, Shanghai, 200000 China; 3 Department of Pharmaceutics, Changhai Hospital, Second Military Medical University, Shanghai, 200433 China

**Keywords:** Hybrid biomimetic membrane, Metformin, FGL1 siRNA, Immunotherapy, Breast cancer

## Abstract

The combination of an immuno-metabolic adjuvant and immune checkpoint inhibitors holds great promise for effective suppression of tumor growth and invasion. In this study, a pH-responsive co-delivery platform was developed for metformin (Met), a known immuno-metabolic modulator, and short interfering RNA (siRNA) targeting fibrinogen-like protein 1 mRNA (siFGL1), using a hybrid biomimetic membrane (from macrophages and cancer cells)-camouflaged poly (lactic-*co*-glycolic acid) nanoparticles. To improve the endo-lysosomal escape of siRNA for effective cytosolic siRNA delivery, a pH-triggered CO_2_ gas-generating nanoplatform was developed using the guanidine group of Met. It can react reversibly with CO_2_ to form Met-CO_2_ for the pH-dependent capture/release of CO_2_. The introduction of Met, a conventional anti-diabetic drug, promotes programmed death-ligand 1 (PD-L1) degradation by activating adenosine monophosphate-activated protein kinase, subsequently blocking the inhibitory signals of PD-L1. As a result, siFGL1 delivery by the camouflaged nanoparticles of the hybrid biomimetic membrane can effectively silence the FGL1 gene, promoting T-cell-mediated immune responses and enhancing antitumor immunity. We found that a combination of PD-L1/programmed death 1 signaling blockade and FGL1 gene silencing exhibited high synergistic therapeutic efficacy against breast cancer in vitro and in vivo. Additionally, Met alleviated tumor hypoxia by reducing oxygen consumption and inducing M1-type differentiation of tumor-related macrophages, which improved the tumor immunosuppressive microenvironment. Our results indicate the potential of hybrid biomimetic membrane-camouflaged nanoparticles and combined Met-FGL1 blockade in breast cancer immunotherapy.
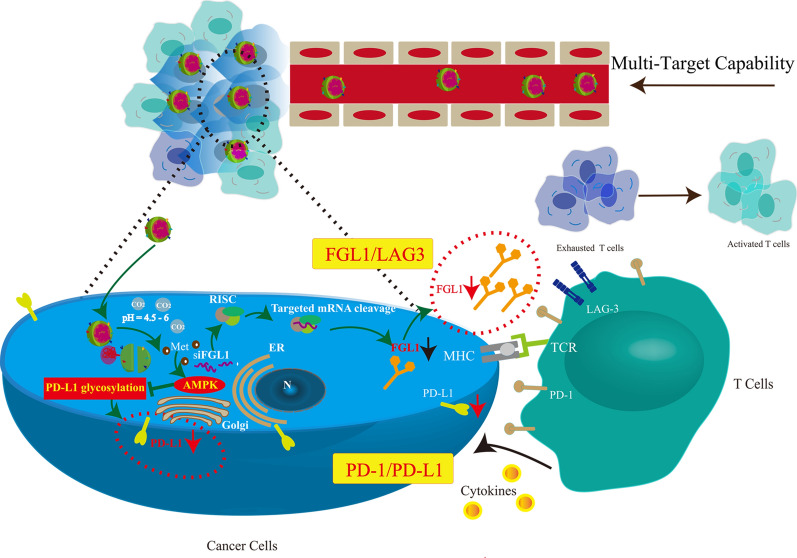

## Background

Immunotherapy is a groundbreaking therapeutic approach that provides unparalleled opportunities to significantly improve the treatment of various diseases, including cancer [1–3]. Distinct from conventional antitumor drugs, which are often harmful to normal cells, immunotherapeutics enable the immune system to recognize and kill tumor cells. However, the immunosuppressed tumor microenvironment and systemic toxicity limit the efficacy and widespread use of immunotherapy [4, 5]. During immunoediting, tumors can escape immune surveillance by activating inhibitory signals, also called immune checkpoints, which are involved in the maintenance of immune homeostasis and self-tolerance [6, 7]. Immune checkpoint inhibitors (ICIs) have shown promise in advancing the treatment of patients with advanced cancers [8].

Programmed death ligand-1 (PD-L1) is a well-studied immune checkpoint inhibitor of programmed death 1 (PD-1), a T-cell negative regulator, and is upregulated on the surface of solid tumors, including breast cancers [2, 9]. PD-L1 plays a critical role in suppressing antitumor immune responses. Blocking the PD-1/PD-L1 interaction exhibits remarkable anticancer therapeutic benefits and is a promising treatment option for the development of new immune checkpoint blockades and combination treatments. Additionally, metformin (Met) has been reported to directly exert antitumor effects by activating adenosine monophosphate-activated protein kinase (AMPK) in the AMPK/mammalian target of rapamycin (mTOR) pathway, which is critical for cancer progression, and inhibiting mitochondrial respiratory-chain nicotinamide adenine dinucleotide (NADH) dehydrogenase (complex I) activity, thus suppressing the respiration of cancer cells [10–12]. Additionally, Met indirectly exerts an antitumor effect by decreasing glucose and insulin metabolism, thereby suppressing tumor cell growth [13, 14]. Furthermore, recent studies have demonstrated that Met can regulate the differentiation and activity of T cells via an intrinsic pathway [15] to promote PD-L1 degradation and activate AMPK, and subsequently block PD-L1/PD-1 signaling [16]. These studies illustrate that the anticancer mechanism of Met is immune-mediated. Thus, Met has the potential to improve the therapeutic outcomes of immunotherapy by blocking PD-L1/PD-1 signaling.

Lymphocyte-activation gene 3 (LAG3) is regarded as one of the main inhibitory receptors associated with depleted T lymphocytes, especially in tumors [17]. Fibrinogen-like protein 1 (FGL1) has been identified as the main functional ligand of LAG3, independent of major histocompatibility complex class II (MHC-II), which is crucial for the negative regulation of T-cell function. Monoclonal antibody-mediated FGL1/LAG3 blockade therapies have proven beneficial in promoting T-cell-mediated immune responses and enhancing antitumor immunity. FGL1 is produced in excess by tumor cells, and the plasma concentration of FGL1 is negatively correlated with the prognosis and resistance to the PD-1/PD-L1 axis blockade [18]. Despite a lack of thorough understanding of the underlying mechanism, several studies have shown that immune checkpoints have non-overlapping functions with unique properties, providing theoretical support for combination therapy with multiple ICIs [19, 20]. Breast cancer is a well-known leading cause of death from malignant tumors in women, and FGL1 is remarkably upregulated in several types of tumors, including breast cancer [21]. In a previous study [22], we demonstrated that delivering PD-L1 small interfering RNA and indoleamine 2, 3-dioxygenase inhibitor could produce synergistic effects against breast cancer. In this regimen a dual blockade of an immune checkpoint was achieved using self-assembled micelles with functional peptides. In the present study, a breast cancer model was used to evaluate the synergistic immunotherapy efficacy of short interfering RNA (siRNA) targeting FGL1 (siFGL1) and Met.

**Scheme 1:**
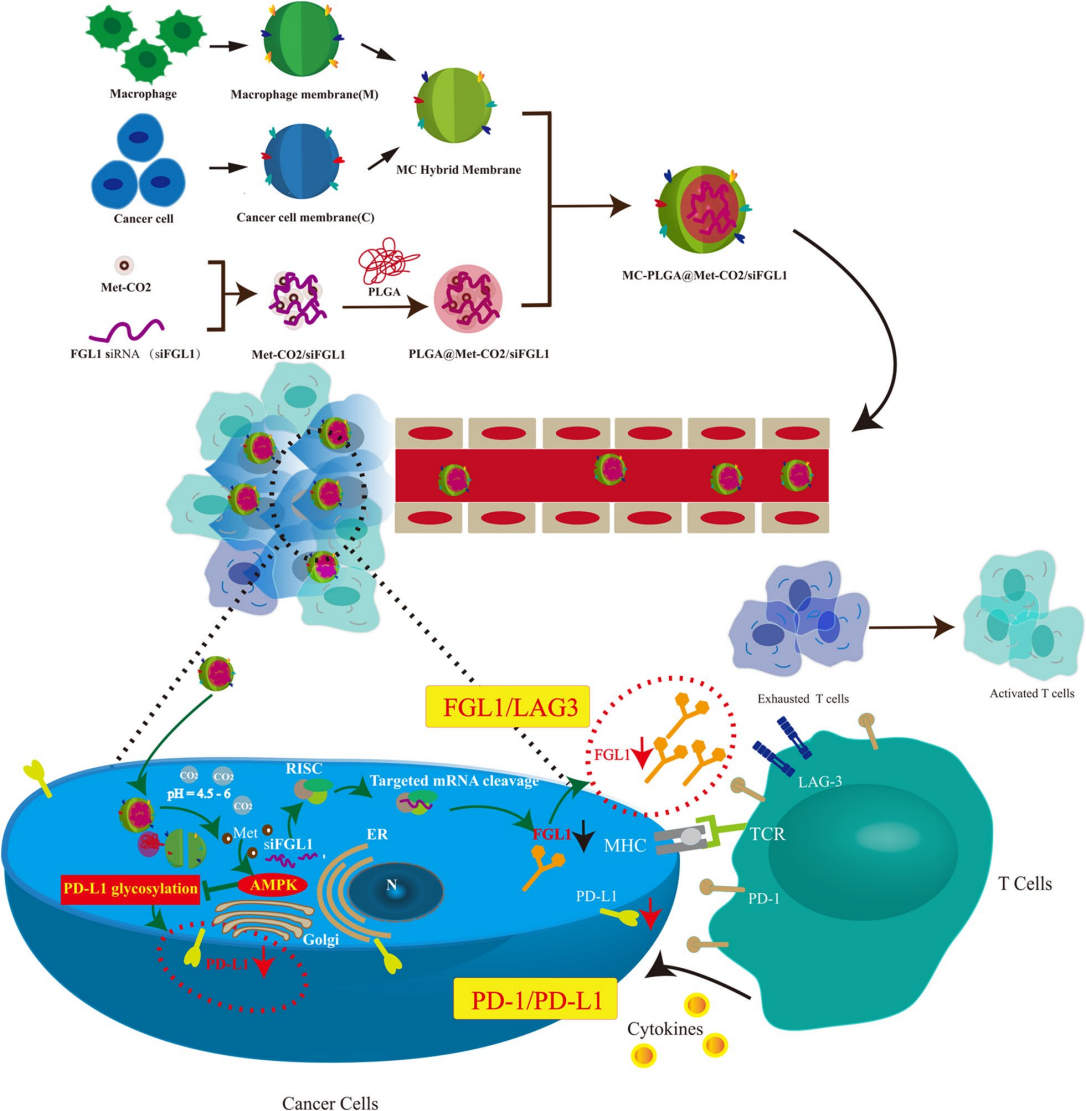
Synthesis and intracellular uptake of metformin and FGL1 siRNA-loaded pH-responsive hybrid membrane-coated nanoparticles (MC-PLGA@Met-CO2/siFGL1 NPs)

In recent years, the cell membrane-based biomimetic nanoparticles (NPs) have been in the spotlight for therapeutic and imaging applications [23–25]. The resulting cell membrane camouflaged NPs preserve the physicochemical properties of the NP core while possessing the complex components of a natural cell membrane, which might endow the NPs with many desirable biological functions. Recently, a variety of cells except red blood cells (RBCs), including platelets, immune cells (e.g., macrophages), and cancer cells have been used to obtain membrane materials [26–29]. Compared with single cell membrane, hybrid cell membrane-coating approach can endow synthetic NPs with multifunctional and complex cell-like functions [30]. Moreover, nano drug co-delivery system has emerged as a promising tool to achieve multi-modal cancer therapy [31]. Recent investigations have supported the synergistic antitumor effects of Met combined with other chemotherapeutic drugs or photosensitizers. The benefits of this combination therapy are typically best realized when the two therapeutic modalities are delivered by single cell membrane- or hybrid cell membrane-coated NPs simultaneously [11, 14, 32]. In this study, we selected a biomimetic nanoplatform using a hybrid membrane-biomimetic poly (lactic-*co*-glycolic acid) (PLGA)-based nanocarrier, as previously reported [33]. We took advantage of the hybrid biomimetic membrane camouflaged core–shell nanoplatform to deliver Met and siFGL1. Met and siFGL1 were encapsulated in PLGA to form a core, which was subsequently covered with a hybrid biomimetic membrane from macrophages and cancer cells to build a multiple-targeting biomimetic nanoplatform. A pH-triggered CO_2_ gas-generating nanoplatform was designed to improve the endosomal/lysosomal escape of the encapsulated siRNA for effective cytosolic siRNA delivery. The guanidine group of Met can react reversibly with CO_2_ to form Met-CO_2_ for the pH-dependent capture/release of CO_2_, which could facilitate low-pH-activated endosomal/lysosomal escape of the encapsulated siRNA. This hybrid biomimetic membrane, obtained by fusing RAW264.7 macrophage membranes (M) and 4T1 breast cancer cell membranes (C), has been proven to possess the multi-targeting capability. We postulated that Met and siFGL1 would exert a synergistic immunotherapy effect. For this purpose, Met and siFGL1-loaded hybrid biomimetic membrane camouflaged nanoparticles (MC-PLGA@Met-CO2/siFGL1 NPs, Scheme 1) were prepared, and their synergistic immunotherapy effects were further investigated in vitro and in vivo*.*

## Methods

### Materials

Metformin (catalog #M258858) was purchased from Toronto Research Chemicals (Toronto, Canada). PLGA (50:50, Mw 35000) was purchased from Lactel Absorbable Polymers (Pelham, AL, USA) and poly (vinyl alcohol) (PVA, 80% hydrolyzed, Mw 9–10 kDa) was purchased from Sigma-Aldrich (catalog #360627-500G). 4′, 6-diamidino-2-phenylindole (DAPI) was obtained from Cayman Chemical Company (Ann Arbor, MI, USA). Annexin V-FITC/PI apoptosis detection reagent was supplied by BD (USA). Bicinchoninic acid (BCA) protein assay reagent kit was purchased from Pierce Chemical Co. (Rockford, IL, USA). PD-L1 antibody (catalog #66248-1-Ig), AMPK-α antibody (catalog #66536-1-lg), FGL1 antibody (catalog #16000-1-AP) were obtained from Proteintech (Chicago, USA). Phospho-AMPK-α (Thr172) (40H9) rabbit monoclonal antibodies (catalog #2535) were obtained from Cell Signaling Technology (Beverly, MA, USA). Rabbit anti-CD86/PE (bs-1035R-PE) and anti-macrophage mannose receptor 1/fluorescein isothiocyanate (FITC) (bs-23178R-FITC) were purchased from Beijing Biosynthesis Biotechnology Co. Ltd (Beijing, China). Negative siRNA (siNeg) control: sense, 5′-UUCUCCGAACGUGUCACGUdTdT-3′ and antisense, 5′-ACGUGACACGUUCGGAGAAdTdT-3′; siFGL1: sense, 5′-GAAGUCCAGUUCCUUGAUAdTdT-3′ and antisense, 5′-UAUCAAGGAACUGGACUUCdTdT-3′; Fluorescein amidite (FAM)-labeled siFGL1 (FAM-siFGL1), and Cy5.5 labeled siFGL1(Cy5.5-siFGL1) were synthesized by GenePharma (Shanghai, China).

### Animals

Animal experiments were conducted in accordance with the United States National Institutes of Health Guide for the Care and Use of Laboratory Animals (NIH Publication No. 8023, revised 1978) and the experiment protocols were approved by the Shanghai Jiao Tong University Animal Care and Use Committee.

### ***Preparation and characterization of MC-PLGA@Met-CO***_***2***_***/siFGL1 NPs***

Before preparing the NPs, Met-CO_2_ was prepared by bubbling an aqueous solution of Met for 1 h with CO_2_. The Met-CO_2_ solution was subsequently lyophilized, ^13^C-nuclear magnetic resonance (^13^C-NMR; 400 MHz) was performed after re-dissolving the samples in D_2_O. Fourier-transform infrared (FTIR) spectra were obtained using KBr windows on a Perkin-Elmer spectrophotometer in the range of 4000–400 cm^−1^. We incorporated both Met-CO_2_ and siFGL1 in PLGA@Met-CO_2_/siFGL1 NPs by adopting the double emulsion (w/o/w) process. In brief, 20 mg of PLGA was dissolved in 2 mL of dichloromethane. This organic solution and 400 µL of deionized (DI) water dissolved in 1 mg/mL Met-CO_2_ and 500 µg/mL FGL1 siRNA (without or with Cy5.5 modification, Sigma) were transferred into a centrifuge tube (50 mL). The sample was then emulsified using a probe-type sonicator at 4 °C for 1 min. A total of 8 mL of the secondary water phase (2.0% w/v PVA) was added to the primary emulsion, and the sample was further emulsified for 1 min. The organic solvent was removed by rotary evaporation at 30 °C, and the rest of the solution was collected by centrifugation for 10 min at 10,000 rpm and washed twice in DI water. The encapsulation efficiency (EE%) of siRNA was calculated as the ratio of the amount of siRNA encapsulated in the NPs to the total amount of siRNA fed for encapsulation. The loading content of the siRNA was calculated as the ratio of the amount of siRNA encapsulated in the NPs to the total amount of NPs including the siRNA. Both the encapsulation efficiency and loading content were quantified using Cy5.5-siFGL1 for encapsulation. Similarly, the encapsulation efficiency and loading content of Met were determined. The amount of Cy5.5-siFGL1 in a sample was determined by fluorescence measurement using a GloMax-Multi Jr Single Tube Multimode Reader (Promega, Madison, WI). The concentration of Met was then determined using a UV spectrophotometer (UV- 2600, Shimadzu, Japan) at 233 nm [34, 35].

The cell membrane was isolated from RAW264.7 and 4T1 cells, and the fused RAW-4T1 hybrid membrane (MC) was prepared in accordance with our previously reported approach [33]. The RAW264.7 cell membrane (M), 4T1 cell membrane (C), or fused RAW-4T1 hybrid membrane (MC) were then coated onto the core of PLGA@Met-CO_2_/siFGL1 NPs by sonicating for 2 min using an FS30D bath sonicator (Fisher Scientific, Waltham, MA) to form the final cell membrane-coated NPs. The MC-PLGA@Met-CO_2_/siFGL1 NPs were maintained at different pH values (pH = 7.4, 6.0, and 5.0) of HEPES buffer, and their size distribution and zeta potential were measured to characterize the components of cell membrane and pH sensitivity by dynamic light scattering (DLS, Zetasizer Nano ZS90, Malvern). The morphology of cell membrane-coated NPs at different pH values (pH = 7.4, 6.0, and 5.0) was also observed by transmission electron microscopy (TEM) (TECNAI G2STWIN, FEI, Hillsboro, Oregon, USA). Additionally, immunogold staining, concomitant with TEM, was conducted to provide visual evidence that the MC hybrid membrane and PLGA@Met-CO_2_/siFGL1 were simultaneously present using both M and C membrane markers, as previously reported [33].

### Agarose gel electrophoresis

To assess the siRNA condensation ability of Met-CO_2_, an appropriate amount of siFGL1 was added to the Met-CO_2_ solution at various W_Met-CO2_/W_siFGL1_ ratios (0, 0.125, 0.25, 0.5, 1, 2, 4, 8, and 10) and incubated for 30 min at room temperature. The solution was then electrophoresed at 100 V for 1 h on a 1% (w/v) agarose gel with Gelred stain in Tris–acetate-EDTA buffer. The gel was subsequently photographed using a UV transilluminator. To investigate whether pH affected the release of siFGL1 from cell membrane-coated NPs, Met-CO_2_-siFGL1, PLGA@Met-CO_2_/siFGL1 NPs, and MC-PLGA@Met-CO_2_/siFGL1 NPs (W_Met-CO2_/W_siFGL1_ = 1) were mixed at different pH values in sodium phosphate buffer for 30 min at room temperature. The samples were then subjected to agarose gel retardation assay. For the RNase A protection assay, the samples of Met-CO_2_-siFGL1, PLGA@Met-CO2/siFGL1 NPs and MC-PLGA@Met-CO_2_/siFGL1 NPs (W_Met-CO2_/ W_siFGL1_ = 0.25) were incubated with RNase A (dose: W_siRNA_/W_RNase A_ = 1: 5 per well) for 30 min. The electrophoretic gel assay was performed as described above.

### Cellular uptake assay

In order to evaluate the cellular uptake efficiency of siFGL1 mediated by MC-PLGA@Met-CO_2_/siFGL1 NPs, FAM-siFGL1 was entrapped into the hybrid membrane-coated NPs. Briefly, 4T1 cells were seeded in 12-well plates at a cell density of 1.5 × 10^5^ cells per well. After culturing for 24 h, the cells were incubated with FAM-siFGL1, PLGA@Met-CO_2_/FAM-siFGL1 NPs, and MC-PLGA@Met-CO_2_/FAM-siFGL1 NPs at 37 °C for up to 4 h. The cellular uptake of FAM-siFGL1 by PLGA NPs with or without hybrid membrane coating was evaluated using a flow cytometer (FACSCalibur; BD Biosciences, UK). The experiment was repeated three times.

Confocal laser scanning microscopy (CLSM) was used to observe the distribution of FAM-siFGL1-mediated by PLGA NPs with or without hybrid membrane coating in cells. Briefly, 4T1 cells were seeded (5 × 10^4^ cells per well) on 24-well plates with a glass bottom. After culturing for 12 h, cells were incubated with FAM-siFGL1, PLGA@Met-CO_2_/FAM-siFGL1 NPs, or MC-PLGA@Met-CO_2_/FAM-siFGL1 NPs at 37 °C for 4 h. The cell culture medium was removed, and the cells were washed thrice with phosphate-buffered saline (PBS) and fixed using 4% paraformaldehyde. The cells were then treated with DAPI for nuclear staining. Finally, the cells were observed by CLSM (Olympus, Tokyo, Japan).

### Intracellular lysosome escape assay

4T1 cells were treated with MC-PLGA@Met/FAM-siFGL1 NPs (without CO_2_), and MC-PLGA@Met-CO_2_/FAM-siFGL1 NPs (with CO_2_) for 2 h and 4 h, respectively, and used with Met instead of Met-CO_2_ for hybrid membrane-coated NPs without CO_2_. The cell culture media was then removed and incubated at 37 °C for 40 min with preheated cell culture medium, with 50 nM of Lyso-Tracker Red (Molecular Probe, Invitrogen Co., OR, USA) for lysosomal staining. Subsequently, the cells were washed thrice with PBS and then treated with DAPI for nuclear staining. Finally, the cells were observed by CLSM (Olympus). In order to further observe the lysosomal escape phenomenon in MC-PLGA@Met-CO_2_/FAM-siFGL1 NPs (with CO_2_), sequential two-dimensional CLSM images were obtained to produce three-dimensional (3D) CLSM images.

### In vitro release of Met

Met release from MC-PLGA@Met-CO_2_/siFGL1 NPs at different pH values (pH = 7.4, 6.0, and 5.0) was investigated by dialysis against PBS at 37 °C. Briefly, 5.0 mL of MC-PLGA@Met-CO_2_/siFGL1 NPs was transferred into a dialysis bag (MWCO 3500 Da), followed by immersion in 30 mL of PBS at different pH values (pH = 7.4, 6.0, and 5.0), with stirring at 120 rpm at 37 °C. At set intervals, 1 mL of external solution was replaced with an equivalent volume of fresh PBS. The concentration of released free Met was then determined using a UV spectrophotometer (UV-2600, Shimadzu, Japan) at 233 nm.

### Cell cycle analysis

The cell cycle was evaluated using a cell cycle staining kit (Multisciences Biotech, Hangzhou, China). Briefly, 4T1 cells were seeded in 12-well plates at a cell density of 1.5 × 10^5^ cells per well. After culturing for 24 h, cells were incubated with Met-CO_2_, MC-PLGA@Met-CO_2_ NPs, and MC-PLGA@Met-CO_2_/siFGL1 NPs at 37 °C for 48 h (Met: 10 mM; siFGL1: 100 nM). The cells were collected and incubated with DNA staining solution and permeabilization solution, in accordance with the manufacturer’s instructions. Finally, the cell-cycle distribution was analyzed using flow cytometry (FACSCalibur; BD Biosciences, Oxford, UK).

### Cytocompatibility assessment

The toxicity of the blank nanomaterials without Met and siFGL1 against 4T1 cells was determined by cell viability assay. Briefly, 4T1 cells (8 × 10^3^ cells per well) seeded into 96-well plates were incubated in 5% CO_2_ at 37 °C. Twentyfour hours later, 100 μL of different concentrations of MC-PLGA NPs (0, 25, 50, 100, 200, 400 µg mL^−1^) were added into each well of the 96-well plate. Twenty-four and 48 h later, 10 μL of CCK-8 was added to each well and and the cells were continued to incubate for 2 h in 5% CO_2_ at 37 °C. The plates were then subjected to a cell viability assay, using a microplate reader at 450 nm. The results were calculated as the means ± SD of at least five independent experiments.

### Tumor cell killing assay mediated by co-culture

To determine the rate of tumor cell apoptosis, 4T1 cells were pretreated using 25 ng/mL of interferon-γ (*IFN-γ*), which was used to stimulate a high expression of PD-L1 [36]. After 24 h, the cells were counted and seeded in 12-well plates and co-cultured with lymphocytes (separated from the spleens of female BALB/C mice for 6 weeks) at a ratio of 1:10 (tumor cell/lymphocyte). The co-cultured cells were then incubated with Met-CO_2_, siFGL1, MC-PLGA@Met-CO_2_ NPs, MC-PLGA@siFGL1 NPs, and MC-PLGA@Met-CO_2_/siFGL1 NPs at 37 °C for 48 h (Met: 10 mM; siFGL1: 100 nM). Finally, lymphocytes and cell debris were removed by PBS, and the cancer cells were collected, stained with an Annexin V-PI apoptosis kit, and detected using flow cytometry.

### Western blot analysis

Western blotting was used to evaluate protein expression of the AMPK signaling pathway in tumor cells influenced by Met. Briefly, 4T1 cells were seeded in 6-well plates at a density of 5 × 10^5^ cells per well. After culturing for 24 h, the cells were incubated with different concentrations of Met delivery by MC-PLGA@Met-CO_2_/siFGL1 NPs at 37 °C for 48 h. In addition, to analyze the knockdown efficiency of siFGL1 and the reduction of PD-L1 levels by Met-activated AMPK, the cells were incubated with different concentrations of MC-PLGA@Met-CO_2_/siFGL1 or MC-PLGA@Met-CO_2_/siNeg NPs at 37 °C for 48 h. The cells were harvested and incubated with radioimmunoprecipitation assay buffer, and the protein concentration of cell lysates was measured using a BCA protein assay kit. The protein sample was transferred to polyvinylidene fluoride membranes, blocked with 5% nonfat milk, and then incubated with primary antibodies at 4 °C for 12 h. Membranes were then washed and incubated with the secondary antibody for 30 min at room temperature. Finally, the membranes were washed and visualized using enhanced chemiluminescence (Millipore, Bedford, MA, USA). β-actin was used as a loading control. Images were obtained using a GE Image Quant Las 4000 mini (GE, Fairfield, CT, USA).

### Antitumor effect in vivo

To investigate the in vivo antitumor effect of MC-PLGA@Met-CO_2_/siFGL1 NPs, 1 × 10^6^ 4T1 cancer cells were injected subcutaneously into the right breast of mice, thereby establishing a 4T1 mouse allograft model of breast cancer. Once the tumor volume reached ∼ 100 mm^3^, the 4T1 murine breast cancer-bearing mice were randomly divided into 6 groups (n = 6, each group), as follows: (1) normal saline control group, (2) Met-CO_2_–treated group, (3) siFGL1-treated group, (4) MC-PLGA@Met-CO_2_ NP-treated group, (5) MC-PLGA@siFGL1 NP-treated group, and (6) MC-PLGA@Met-CO_2_/siFGL1 NP-treated group, with the Met equivalent of 16 mg/kg and siFGL1 of 2 mg/kg. The drugs were injected via the tail vein every four days, four consecutive times. Tumor volumes and animal body weights were monitored regularly. The tumor volume was calculated according to the following formula: (length × width^2^/2). The survival period was also recorded. After completion of the experiment, tumors were excised and weighed. The tumor inhibitory rate (TIR) was calculated using the following equation: TIR = [1 − (W_t_ − W_i_)/ (W_s_ − W_i_)] × 100%, where W_t_ is the final mean tumor weight of the tested groups, W_i_ is the initial mean tumor weight of the tested groups, and W_s_ is the final mean tumor weight of the saline group.

### Flow cytometry analysis and ELISA

Tumor specimens were cut into small pieces with a scissor. After lysis of red blood cells using red blood cell lysis buffer, the resulting cells were filtered through nylon-mesh filters (70 μm). The single-cell suspensions were re-suspended in PBS containing 2% fetal bovine serum. The cell suspension was stained with anti-CD3-FITC, anti-CD4-PE, and anti-CD8-APC antibodies, following the manufacturer’s protocols to analyze the T cells. The cells were then analyzed using flow cytometry. We also analyzed the levels of tumor-associated *IFN-γ* and interleukin-2 (*IL-2*) in tumor samples obtained from the mice after treatment using an enzyme-linked immunosorbent assay (ELISA), following the manufacturer’s protocol. Furthermore, the tumor tissue sections were stained with Ki-67 and hematoxylin and eosin (H&E) for histopathologic analysis.

### Immunofluorescence

Tumor tissues collected from sacrificed mice were fixed in 4% paraformaldehyde solution and embedded in paraffin for immunofluorescence staining. We assessed the hypoxic area of the tumor tissues in each group using Hypoxyprobe™-1 Plus Kits (FITC- Mab, Hypoxyprobe, Inc., Burlington MA) as a hypoxia probe. To observe the effect of MC-PLGA@Met-CO_2_/siFGL1 NPs on the polarization of tumor-associated macrophages (TAMs), rabbit polyclonal FITC-anti-macrophage mannose receptor 1 antibody (dilution 1:50) and rabbit polyclonal PE-CD86 antibody (dilution 1:50) were used to determine the distribution of macrophages with M1 or M2 phenotype in tumor tissues, respectively. CD8 positive cells were stained with anti-mouse CD8 antibody and detected by Cyanine 3 (Cy3)-labeled secondary antibody. Nuclei were stained with DAPI. Finally, the sections were observed under CLSM (Zeiss LSM710) and all images were digitally acquired using LSM soiware (ZEN2010).

### Histology and immunohistochemistry analysis

Histological examination of the major organs (the heart, liver, spleen, lung, and kidney) and tumor tissues was performed using H&E staining. At the end of the treatment, the major organs and tumor tissues were collected and immediately fixed in 4% paraformaldehyde for one day, embedded in paraffin, and then sectioned. The major organs were stained with H&E. Moreover, the tumor issue sections were immunohistochemically stained for PD-L1, FGL1, Thr172, and AMPK-α histopathologic analysis.

### Statistical analysis

Data are expressed as the mean ± standard deviation (SD). Analysis of variance (ANOVA) was used to evaluate the differences among groups. A *p*-value < 0.05 was considered statistically significant.

## Results

### ***Synthesis and characterization of MC-PLGA@Met-CO***_***2***_***-siFGL1 NPs***

**Fig. 1:**
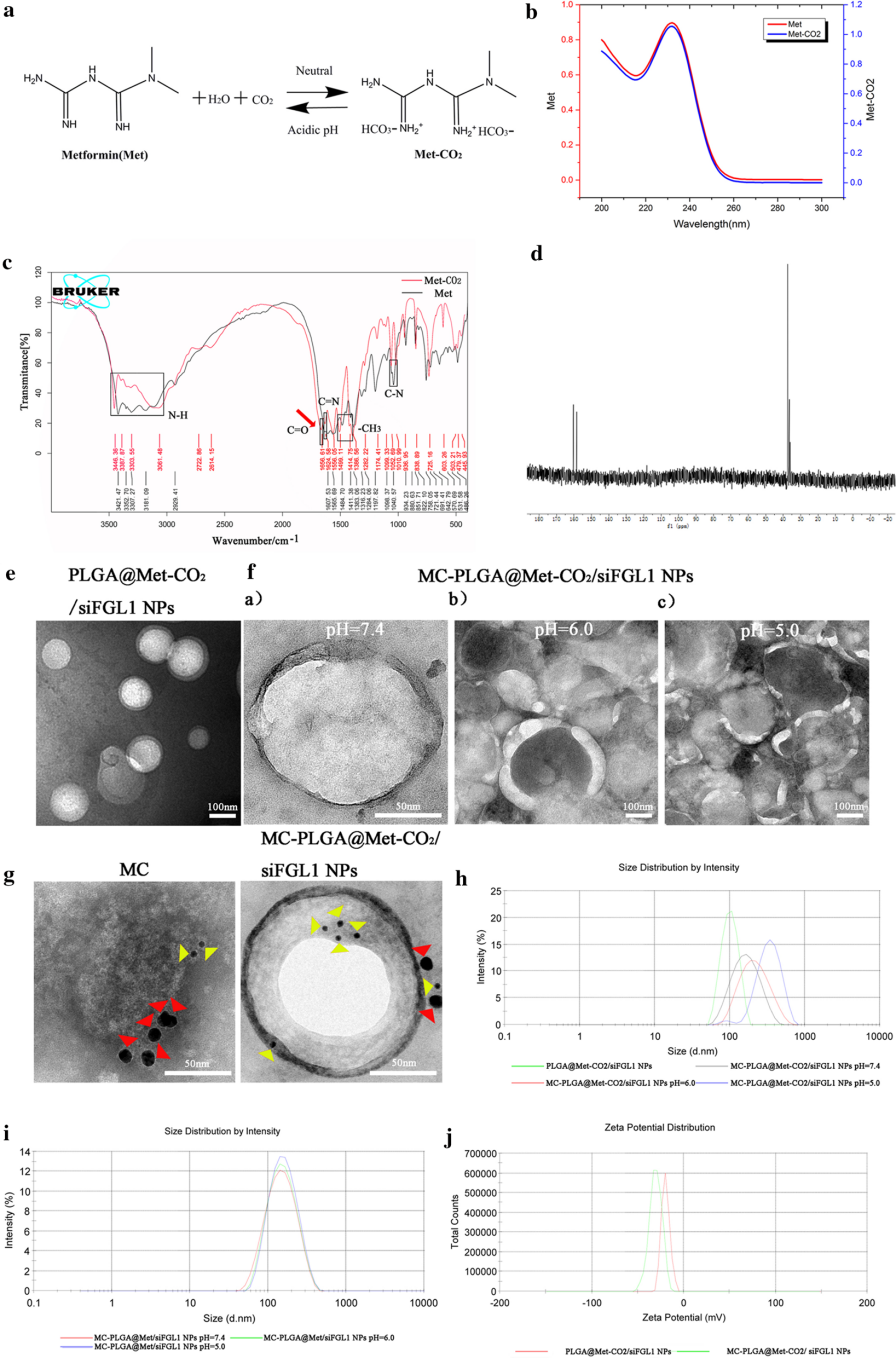
Characterization of MC-PLGA@Met-CO_2_/siFGL1. **a** Illustrations of the chemical reactions for the modification of Met with guanidine and the pH-triggered CO_2_ gas generated by the guanidine-modified Met. **b** UV absorption spectra of Met and Met-CO_2_ in the range of 220–300 nm. **c** FTIR spectra of Met and Met-CO_2_. **d** The ^13^C-NMR spectrum of Met and Met-CO_2_ dissolved in D_2_O. **e** Representative TEM images of PLGA@Met-CO_2_/siFGL1 NPs negatively stained with vanadium (scale bar = 100 nm). **f** Representative TEM images of MC-PLGA@Met-CO_2_/siFGL1 NPs at pH 7.4 (a, scale bar = 50 nm), 6.0 (b, scale bar = 100 nm), and 5.0 (c, scale bar = 100 nm), negatively stained with vanadium. **g** Immunogold TEM images of MC and MC-PLGA@Met-CO_2_/siFGL1 NP samples probed for α4 (red arrows, large gold) and vascular cell adhesion molecule-1 (yellow arrows, small gold) after negative staining with 2% sodium phosphotungstate (scale bar = 50 nm). **h** NP size distribution of PLGA@Met-CO_2_/siFGL1 NPs and MC-PLGA@Met-CO_2_/siFGL1 NPs at pH 7.4, 6.0, and 5.0, determined by DLS. **i** NP size distribution of MC-PLGA@Met/siFGL1 NPs (without CO_2_) at pH 7.4, 6.0, and 5.0, determined by DLS. **j** Zeta potential of PLGA@Met-CO_2_/siFGL1 NPs and MC-PLGA@Met-CO_2_/siFGL1 NPs

Met-CO_2_ was produced by bubbling an aqueous solution of Met for 1 h with CO_2_. As shown in Fig. 1b, both Met and Met-CO_2_ exhibited maximum absorbance at about 233 nm; accordingly, the concentrations of both Met and Met-CO_2_ were determined using a UV-spectrophotometer at 233 nm. The formation of Met-CO_2_ was verified using FTIR spectra and ^13^C-NMR. The formation of bicarbonate of Met by CO_2_ are shown by the FTIR transmittance patterns of Met-CO_2_ (red curve) along with Met (black curve) (Fig. 1c). The chemical building of Met-CO_2_ displayed a peak at 1656 cm^−1^, corresponding to the carbonyl absorbing peak (–C=O, red arrow), indicating the formation of bicarbonate of Met by CO_2_. However, the FTIR spectrum of Met-CO_2_ was similar to that of Met. Both Met-CO_2_ and Met displayed peaks at 3446 cm^−1^, 3387 cm^−1^, 3303 cm^−1^, and 3061 cm^−1^, corresponding to the amide groups (amide stretching vibration); at 1671 cm^−1^, corresponding to the imine (C=NH) bond of the Met-CO_2_ or Met; and at 1484 cm^−1^ and 1411 cm^−1^, which belonged to the stretching vibration mode of the two methyl groups attached to one nitrogen atom in Met-CO_2_ or Met. The C-N stretching vibration mode in Met-CO_2_ or Met was located at 1052 cm^−1^. Figure 1d shows the ^13^C-NMR (D_2_O, 400 MHz) spectra of Met-CO_2_. A peak centered at 158.46 ppm emerged, consistent with the formation of carbonate [37]. The spectrum showed characteristic signals for bi-guanidine at 160.24 ppm. Two signals at 37.45 and 36.67 ppm corresponded to the two methoxy carbons in Met-CO_2_.

Representative TEM images of the PLGA@Met-CO_2_/siFGL1 NPs, and MC-PLGA@Met-CO_2_/siFGL1 NPs (pH 7.4, 6.0, and 5.0) are shown in Fig. 1e, f. The MC-PLGA@Met-CO_2_/siFGL1 NPs displayed characteristic core–shell-like bilayer membrane structures. The hybrid membrane-coated NPs were stable at neutral pH of 7.4 (Fig. 1f a) and were 142 ± 3.5 nm in diameter, with a polydispersity index (PDI) of 0.257 ± 0.0203 (Fig. 1h) and negatively charged surface (surface zeta potential, -30.5 ± 2.5 mV; Fig. 1j). The size of the hybrid membrane-coated NPs synthesized using Met without CO_2_ (MC-PLGA@Met/siFGL1 NPs) was not significantly affected at a pH of 6.0 or 5.0 (Fig. 1i). In contrast, when Met-CO_2_ was used, their size increased to 225.7 ± 10.2 nm, and deformation (Fig. 1f b) was observed on some of the hybrid membrane-coated NPs at pH 6.0. When the pH was further reduced to 5.0, the size increased by more than 1.51 times (to 340.9 ± 19.5 nm) for most NPs (Fig. 1h), and defects were observed on all hybrid membrane-coated NPs (Fig. 1f c). Additionally, immunogold labeling, followed by TEM imaging, provided visual evidence that single MC-PLGA@Met-CO_2_/siFGL1 NPs simultaneously presented both 4T1-specific and RAW-specific markers (Fig. 1g). The drug loading (DL) and encapsulation efficiency (EE) for siFGL1 (2.13%, 80.14%) and Met (1.61%, 78.42%) in MC-PLGA@Met-CO2/siFGL1 NPs.

### Agarose gel electrophoresis

**Fig. 2:**
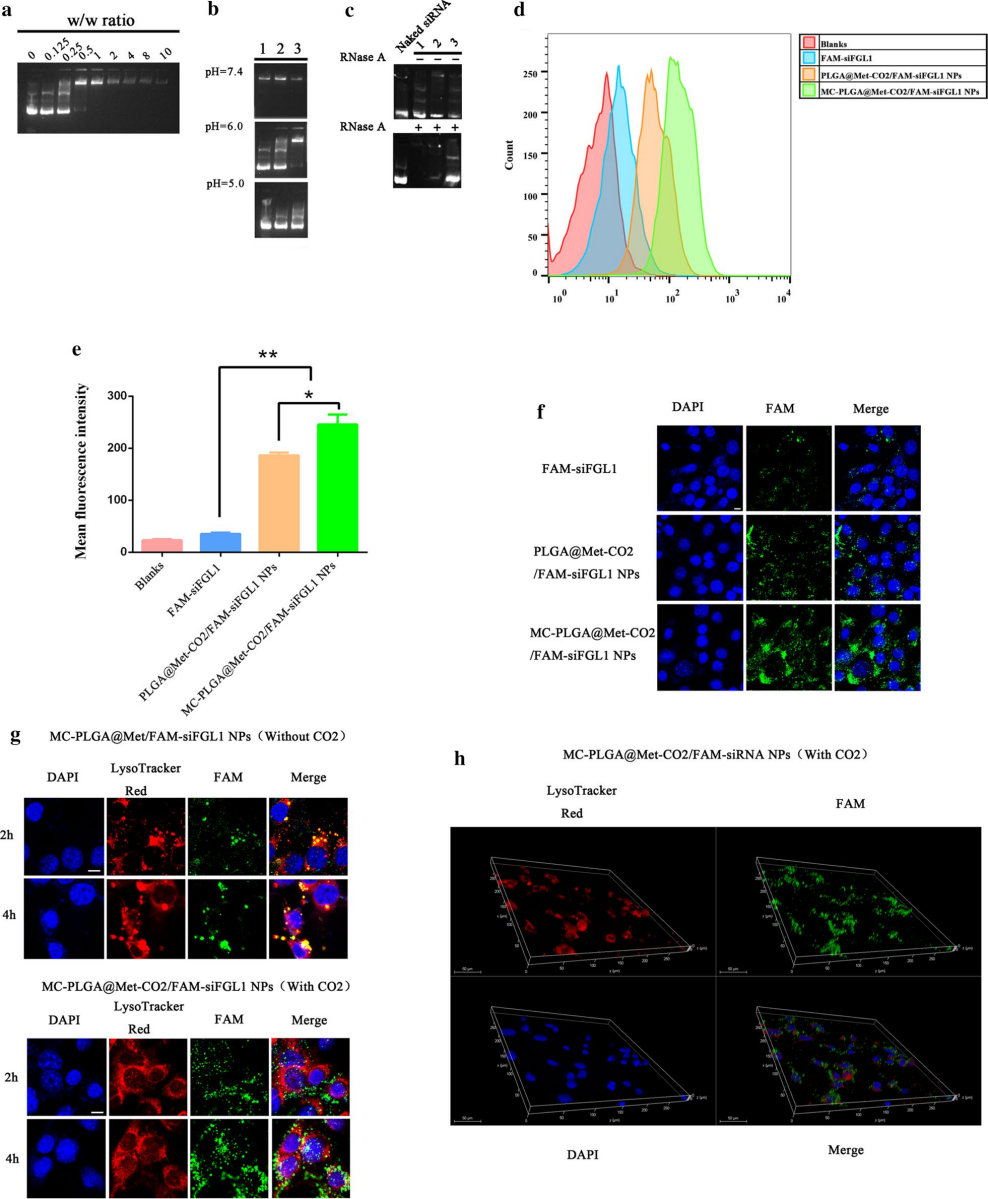
Electrophoretic gel assay, cellular uptake, and endosome escape of MC-PLGA@Met-CO_2_/FAM-siFGL1 NPs in 4T1 cells. **a** Agarose gel electrophoresis of siFGL1 and Met-CO_2_ binding affinity under different N/P ratios. **b** Electrophoretic gel assay of Met-CO_2_-siFGL1 (lane 1), PLGA@Met-CO_2_/siFGL1 (lane 2), and MC-PLGA@Met-CO_2_/siFGL1 (lane 3) (w/ w ratio = 1) at different pH values (pH = 7.4, 6.0 and 5.0). **c** RNase protection assay. Naked siRNA, siRNA encapsulated within the Met-CO_2_-siFGL1 + RNase A (or without adding RNase A) (lane 1), PLGA@Met-CO_2_/siFGL1 + RNase A (or without adding RNase A) (lane 2), MC-PLGA@Met-CO_2_/siFGL1 + RNase A (or without adding RNase A) (lane 3). **d** Flow cytometry analysis of the amount of FAM-siFGL1 internalized by 4T1 cells after 4 h of incubation. **e** Quantitative analysis of the relative fluorescence intensity of 4T1 cells (n = 3). **f** Confocal microscopy images of 4T1 cells incubated with FAM-siFGL1, PLGA@Met-CO_2_/FAM-siFGL1 NPs, or MC-PLGA@Met-CO_2_/FAM-siFGL1 NPs for 4 h. **g** Representative confocal microscopy images of 4T1 cells incubated with MC-PLGA@Met/FAM-siFGL1 NPs (without CO_2_), MC-PLGA@Met-CO_2_/siFGL1 NPs (with CO_2_) for 2 h and 4 h at 37 °C. The cell nuclei were stained using DAPI (blue), the endo/lysosomes were stained using LysoTracker Red (red), and siFGL1 was labeled with FAM (green). The scale bar is 20 μm. **h** Three-dimensional confocal images of 4T1 cells incubated with MC-PLGA@Met-CO_2_/FAM-siFGL1 NPs showing intracellular endosome escape behavior, with green representing siFGL1, red representing endosomes/lysosomes, and blue representing 4T1 cell nuclei

The condensation ability of the complexes was determined by agarose gel electrophoresis. A complete mobility shift of siFGL1 was achieved at a W_Met-CO2_/W_siFGL1_ ratio of 1 (Fig. 2a). The migration of siFGL1 into the electrophoretic agarose gel was almost completely inhibited by Met-CO_2_ or nanoparticle encapsulation, with negligible release at pH 7.4 (Fig. 1b). At pH 6.0, a smaller electrophoresis band was observed, indicating a slower release of the siRNA, while a strong electrophoresis band was observed at pH 5.0 for MC-PLGA@Met-CO_2_/siFGL1 NPs. These data show that the low-pH-trigged nanobomb effect of the MC-PLGA@Met-CO_2_/siFGL1 NPs could result in a pH-sensitive release of the encapsulated siFGL1. Furthermore, to verify whether MC membrane cloaking would protect the siRNA from RNase A degradation, RNase A was added [38]. As shown in Fig. 2c, after incubation with RNase A for 30 min at 37 °C, a bright band still emerged on electrophoresis for MC-PLGA@Met-CO_2_/siFGL1 NPs, whereas the electrophoresis band for Met-CO_2_-siFGL1 and PLGA@Met-CO_2_/siFGL1 NPs had disappeared, which is different from those of Met-CO_2_-siFGL1, PLGA@Met-CO_2_/siFGL1 NPs and MC-PLGA@Met-CO_2_/siFGL1 NPs at 30 min incubation time without RNase A. It was concluded that the MC membranes protected the siRNA from RNase A degradation in the negatively charged PLGA NPs and the guanidine group of Met could condense and protect siRNA, which is consistent with previous reports [39, 40].

### Cellular uptake assay

We further investigated the uptake of FAM-siFGL1 mediated by hybrid membrane-coated NPs using flow cytometry. As shown in Fig. 2d, there was a higher level of uptake of MC-PLGA@Met-CO_2_/FAM-siFGL1 NPs than of PLGA@Met-CO_2_/FAM-siFGL1 NPs, and the difference in relative fluorescence intensity was significant after 4 h (Fig. 2e, p < 0.05). However, hardly any cell uptake was observed in free FAM-siFGL1, which indicates that FAM-siFGL1 cannot be taken up by cells unless it is loaded using the hybrid membrane-coated NPs or PLGA NPs (*p* < 0.01).

CLSM was conducted to observe the intracellular distribution of FAM-siFGL1 mediated by hybrid membrane-coated NPs in 4T1 cells. As shown in Fig. 2f, nuclei stained with DAPI showed green fluorescence, corresponding to FAM-siFGL1. After 4 h of treatment, there was a stronger green fluorescence for MC-PLGA@Met-CO_2_/FAM-siFGL1 NPs than for PLGA@Met-CO_2_/FAM-siFGL1 NPs, while hardly any green fluorescence was observed for free FAM-siFGL1. These results reflected the results obtained from flow cytometry.

### Intracellular lysosome escape assay

To investigate intracellular trafficking, FAM-siFGL1 was encapsulated in MC-PLGA@Met/FAM-siFGL1 NPs (without CO_2_) or MC-PLGA@Met-CO_2_/FAM-siFGL1 NPs (with CO_2_). We labeled intracellular acidic lysosomes with LysoTracker Red, which presents with red fluorescence. Additionally, the location of nuclei is indicated by blue fluorescence, green fluorescence corresponds to FAM-siFGL1, and yellow fluorescence corresponds to the overlap between green and red fluorescence, representing FAM-siFGL1 trapped within the endosome. As shown in Fig. 2g, most FAM-siFGL1 mediated by MC-PLGA@Met/FAM-siFGL1 NPs (without CO_2_) was trapped in the lysosome at both 2 h and 4 h, while FAM-siFGL1 mediated by MC-PLGA@Met-CO_2_/FAM-siFGL1 NPs (with CO_2_) was always distributed outside the lysosome after 2 h or 4 h. We further investigated the intracellular distribution of FAM-siFGL1 mediated by MC-PLGA@Met-CO_2_/FAM-siFGL1 NPs (with CO_2_) using 3D CLSM images. As shown by the merged images in Fig. 2h, the intracellular green fluorescence and red fluorescence did not overlap after 4 h, which further illustrated that CO_2_ facilitates the escape of FAM-siFGL1 from the lysosomes.

### In vitro drug release

**Fig. 3:**
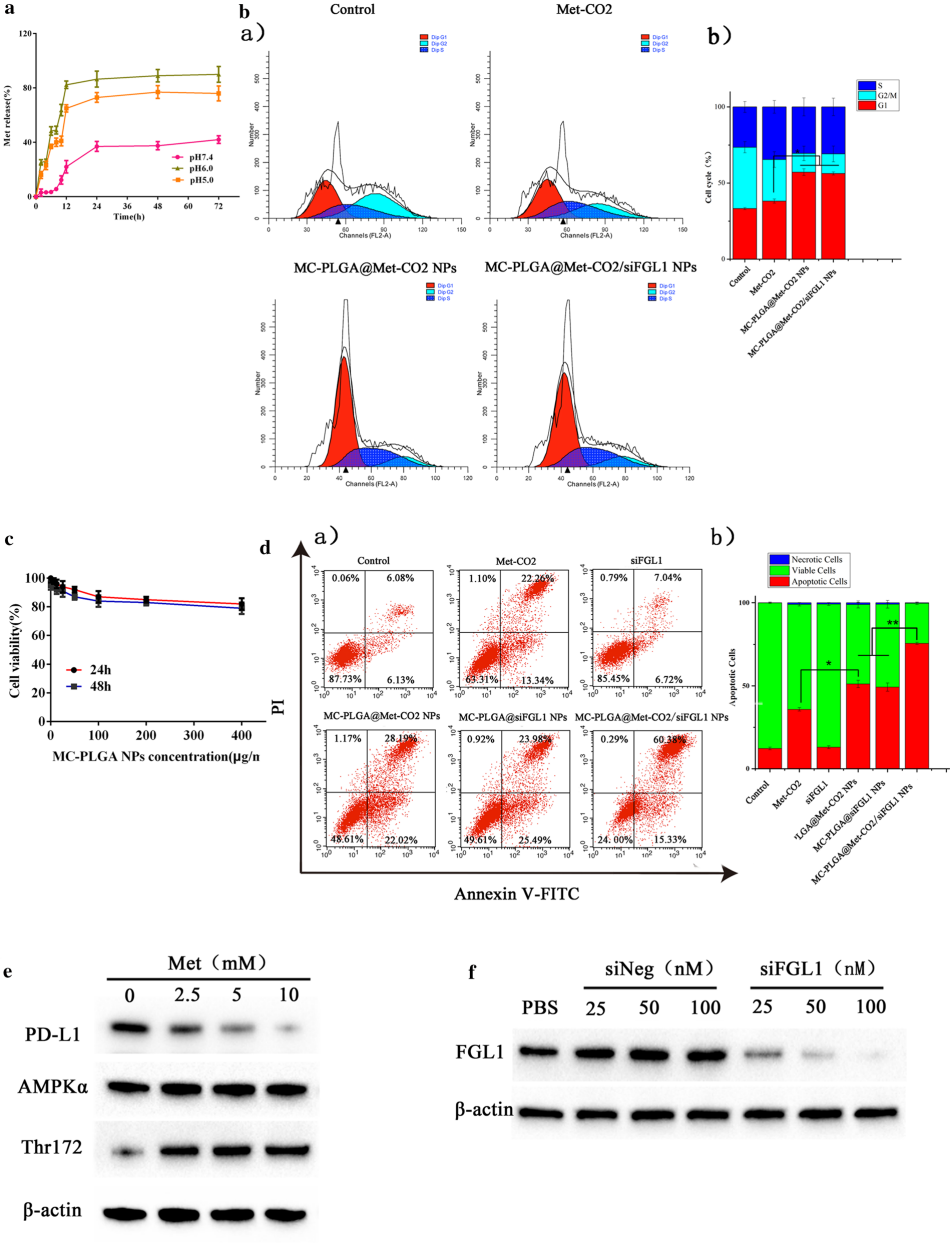
Release of Met from MC-PLGA@Met-CO_2_/FAM-siFGL1 NPs and cytotoxicity evaluation. **a** Release kinetics of Met from MC-PLGA@Met-CO_2_/FAM-siFGL1 NPs at different pH values (pH values of 7.4, 6.0, and 5.0). **b** After treatment with 10 mM Met mediated by various formulations for 48 h, cell cycles of 4T1 cells were examined using flow cytometry assay, and the graph depicts the quantification analysis for each group. **c** The biocompatibility of MC-PLGA NPs. **d** Apoptosis analysis of 4T1 cells after 48 h treatment with different formulations in the co-culture as evaluated by flow cytometry using Annexin V- FITC kit and PI staining. Corresponding statistics on the percentage of and necrotic cells are presented. **e** Western blot analysis of PD-L1, AMPK-α, Thr172 protein levels in 4T1 cells after 48-h treatment with different concentrations of Met mediated by MC-PLGA@Met-CO_2_/siFGL1 NPs. **f** Western blot analysis of FGL1 protein levels in 4T1 cells after 48-h treatment with different concentrations of siFGL1 mediated by MC-PLGA@Met-CO_2_/siFGL1 NPs

The release profiles of Met from the hybrid membrane-coated NPs were investigated at various pH values (pH = 7.4, 6.0, and 5.0) (Fig. 3a). In the first 6 h, nearly 37% and 48% of Met was released at pH 5.0 and 6.0, respectively. MC-PLGA@Met-CO_2_/siFGL1 NPs exhibited a slow drug release of 3% within 6 h at pH 7.4. This result indicates that the hybrid membrane-coated NPs might be more stable under physiological conditions. Met was released faster at pH 5.0 than that at pH 6.0, with approximately 86% and 73%, respectively, of the total Met content released from the hybrid membrane-coated NPs within 24 h. MC-PLGA@Met-CO_2_/siFGL1 NPs released about 37% of Met within 24 h at pH 7.4.

### ***Effects of MC-PLGA@MetCO***_***2***_***/siFGL1 on the cell cycle***

Flow cytometry was used to further investigate the effect of Met on the cell cycle. The effects of Met formulations on cell cycle arrest in 4T1 cells are shown in Fig. 3b. Compared to that with MC-PLGA@Met-CO_2_ NPs, there was a non-obvious upsurge in the accumulation of G1 phase cells among 4T1 cells with MC-PLGA@Met-CO_2_/siFGL1 NPs. This indicated that siFGL1 had no obvious cell cycle arresting effect on 4T1 cells.

### Cytocompatibility assessment

We investigated the toxicity of the blank nanomaterials (MC-PLGA NPs) (Fig. 3c). It was biocompatible during 0–400 μg/mL with the cells viability above 85% after 24- and 48-h incubation with MC-PLGA NPs, indicating a good biocompatibility of the NPs.

### Tumor cell killing assay mediated by co-culture

To further investigate the antitumor response in the co-culture system, flow cytometry was used to analyze cell apoptosis after incubation with the Annexin V-FITC/PI kit. As shown in Fig. 3d a, an increase in cell apoptosis in 4T1 cells was detected with Met-CO_2_ and MC-PLGA@Met-CO_2_ NPs. MC-PLGA@Met-CO_2_ NPs induced cell apoptosis by 50.21%, which was approximately 1.41-fold higher than that for Met-CO_2_. MC-PLGA@Met-CO_2_/siFGL1 NPs induced 4T1 cell apoptosis more effectively than MC-PLGA@Met-CO_2_ NPs, by approximately 1.51-fold. Additionally, siFGL1 failed to induce cell killing efficacy when compared with the control (apoptosis rate: 13.76% vs. 11.21%), which might be attributed to its instability and easy degradation in serum, and limited membrane permeability [41, 42]. MC-PLGA@siFGL1 NPs were also able to induce cell apoptosis in 4T1 cells at a rate of approximately 60.78%, indicating that siFGL1 loaded with the aid of hybrid membrane-coated NPs could silence the FGL1 gene in 4T1 cells, thereby promoting T-cell immunity. Furthermore, MC-PLGA@Met-CO_2_/siFGL1 NPs caused a high rate of apoptosis in 4T1 cells (approximately 75.71%) (*p* < 0.01 vs. MC-PLGA@Met-CO_2_ NPs and *p* < 0.01 vs. MC-PLGA@siFGL1 NPs), indicating that the delivery of Met-CO_2_ NPs and siFGL1 mediated by hybrid membrane-coated NPs had a synergistic antitumor immune killing effect, causing a higher level of tumor cell apoptosis.

### Western blot analysis

Some previous studies indicated that Met possesses improved antitumor immune effects via endoplasmic reticulum-associated degradation of PD-L1 with AMPK activation [16]. Additionally, Met may activate AMPK by causing stress conditions, including mitochondrial dysfunction, which depletes ATP [43]. In many cases, AMPK is regarded as a tumor suppressor [44]. The expression of related proteins in cells was analyzed using western blotting. As shown in Fig. 3e, MC-PLGA@Met-CO_2_/siFGL1 NPs significantly inhibited the expression of PD-L1 in 4T1 cells, with the highest effect observed at a concentration of 10 mM of Met. Simultaneously, the phosphorylation levels of AMPK-α in 4T1 cells were significantly enhanced than that for the control, with the highest effect observed at a concentration of 2.5 mM of Met (Fig. 3e). Moreover, the upregulation of FGL1 is related to a poor prognosis after treatment in several cancers, including breast cancer [18, 21]. Importantly, FGL1 western blotting (Fig. 3f) demonstrated that MC-PLGA@Met-CO_2_/siFGL1 NPs could suppress the expression of FGL1 in 4T1 cells as the concentration of siFGL1 continued to rise to 100 nM.

### Antitumor effect in vivo

**Fig. 4:**
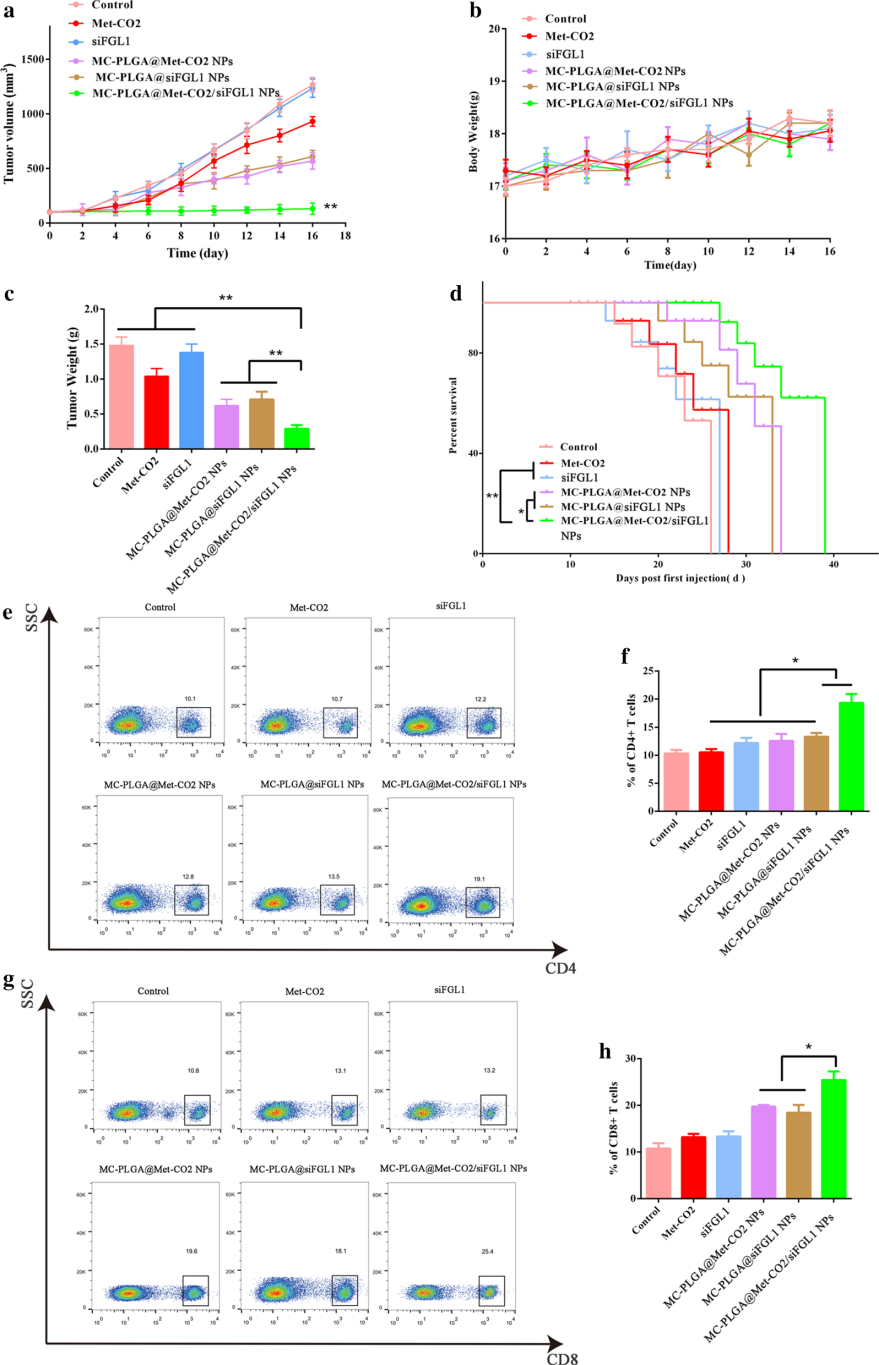
In vivo antitumor effect. **a** Tumor growth profiles during the experimental period of 4T1 tumor-bearing mice in different groups. **b** Bodyweight of 4T1 tumor-bearing mice under different treatments. **c** The tumor weight at the end of the experiment. **d** Survival rate of 4T1 tumor-bearing mice. **e** Representative flow cytometry plots of CD4^+^ T cells by different treatments. **f** Quantitative analysis of the percentage of CD4^+^ T cells. **g** Representative flow cytometry plots of CD8^+^ T cells by different treatments. **h** Quantitative analysis of the percentage of CD8^+^ T cells. The mean ± standard deviation is reported (n = 6) *p < 0.05, **p < 0.01, ***p < 0.001

After showing excellent activity in vitro, different nano-formulations were examined using BALB/c mice bearing 4T1 mammary tumors. The tumor volumes and mouse weights were monitored at the indicated time points (Fig. 4a). As Fig. 4a shows, tumors in the saline-treated and siFGL1-treated groups grew rapidly, with the average tumor volumes reaching over 1235 mm^3^ within 16 days. The tumor volume increased to a greater extent in the MC-PLGA@Met-CO_2_ NP-treated and MC-PLGA@siFGL1 NP-treated groups than in the MC-PLGA@Met-CO_2_/siFGL1 NP-treated group (*p* < 0.01). The overall inhibitory rate by tumor volume was 2.66%, 56.1%, and 97.3% in the siFGL1-treated, MC-PLGA@siFGL1 NP-treated, and MC-PLGA@Met-CO_2_/siFGL1 NP-treated groups, respectively, which illustrates the strong synergism due to the MC hybrid biomimetic membrane-camouflaged PLGA NP-mediated delivery. These in vivo results are consistent with the in vitro results. As shown in Fig. 4b, none of the groups had a significant loss of body weight, demonstrating that the systemic toxicity of our therapeutic regimen was low. The weight of tumor tissues collected from mice on day 16 showed a similar trend in tumor volume change (Fig. 4c). As shown in Fig. 4d, the survival period of mice in the Met-CO_2_-treated and siFGL1-treated groups was 28 days and 27 days, respectively, vs. 39 days in the MC-PLGA@Met-CO_2_/siFGL1 NP-treated group (*p* < 0.01), indicating that MC-PLGA@Met-CO2/siFGL1 NPs effectively inhibited tumor growth and prolonged the survival of mice. Treatment with MC-PLGA@Met-CO_2_ NPs and MC-PLGA@siFGL1 NPs showed slightly improved survival rates, whereas co-loaded MC-PLGA@Met-CO_2_/siFGL1 NPs enhanced the survival period by nearly 40 days (*p* < 0.05).

### Flow cytometry analysis and ELISA

To evaluate the anticancer mechanisms of MC-PLGA@Met-CO_2_/siFGL1 NPs, we further collected tumor-infiltrating lymphocytes to analyze the changes in the specific cellular composition of each tumor by flow cytometry. Compared with MC-PLGA@Met-CO_2_ NPs and MC-PLGA@siFGL1 NPs, MC-PLGA@Met-CO_2_/siFGL1 NPs increased the ratio of CD8^+^ T cells to CD4^+^ T cells (*p* < 0.05) (Fig. 4e–h).

**Fig. 5:**
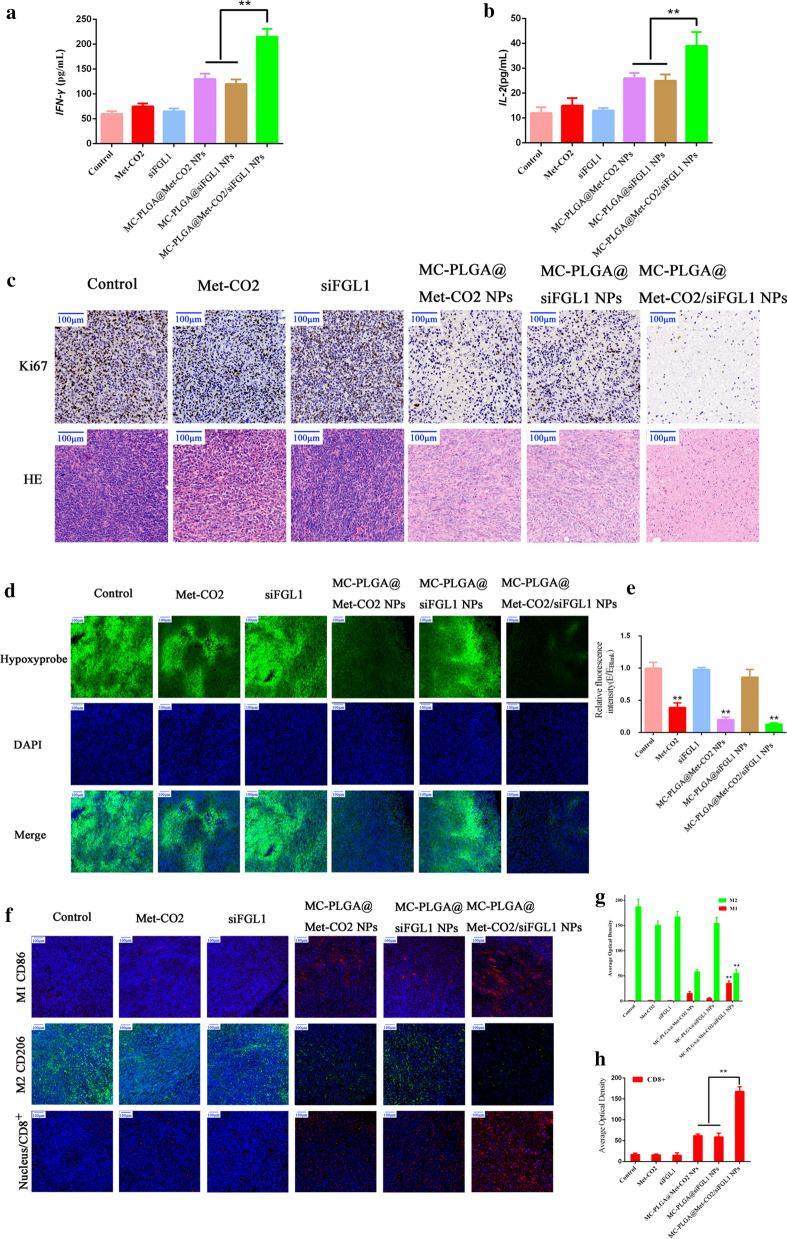
Remodeling of the tumor immune microenvironment. Intratumoral levels of cytokines *IFN-γ* (**a**) and *IL-2* (**b**) as determined by ELISA. **c** Representative images of Ki-67-stained (scale bar, 100 µm) and H&E-stained (scale bar, 100 µm) tumor tissue. **d** Representative immunofluorescence images of tumor slices stained with a hypoxia maker. **e** Quantitative analysis of the hypoxia marker (histogram). **f** Representative immunofluorescence images of tumor slices stained with CD86 (M1, red), CD206 (M2, green), and CD8 (red), respectively; the nucleus was stained with DAPI (blue). Corresponding quantitative analysis (histogram) of the M1 and M2 (**g**) and CD8 T cells (**h**)

The immune response was also investigated by analyzing intratumoral cytokine levels using ELISA. The production of cytokines, including *IFN-γ* and *IL- 2*, which are important biomarkers with a profound influence on the immune response, was determined. As shown in Fig. 5a, b, the cytokine levels were significantly higher in MC-PLGA@Met-CO_2_/siFGL1 NPs group than in MC-PLGA@Met-CO_2_ NPs and MC-PLGA@siFGL1 NPs groups (*p* < 0.01).

Further, H&E and Ki-67 staining analyses were used to evaluate the antitumor effects of the different treatments. In the MC-PLGA@Met-CO_2_/siFGL1 NP-treated group, extensive nuclear pyknosis and cancer necrosis were observed on the tumor, indicating severe cancer necrosis (Fig. 5c). Furthermore, compared to the other groups, the MC-PLGA@Met-CO_2_/siFGL1 NP-treated group showed decreased expression of Ki-67 in tumor tissues, indicating a remarkable decrease in tumor cell proliferation. Collectively, these results indicate the synergistic immunotherapy efficacy of siFGL1 and Met.

### Immunofluorescence

Hypoxyprobe™-1, a substituted 2-nitroimidazole with a chemical name of pimonidazole hydrochloride, is widely used as a specific hypoxic cell marker [45–47]. The immunohistochemistry results showed that the expression areas for Hypoxyprobe-1 (Fig. 5d) were smaller with MC-PLGA@Met-CO_2_ NPs, and MC-PLGA@Met-CO_2_/siFGL1 NPs than with the control (*p* < 0.01). As indicated in Fig. 5f, there was a higher expression of CD86 (M1-phenotype TAMs) and lower expression of CD206 (M2-phenotype TAMs) in MC-PLGA@Met-CO_2_ NPs group and MC-PLGA@Met-CO_2_/siFGL1 NPs group than with the control (*p* < 0.01).

To further evaluate the local immune response, an immunofluorescence staining analysis of tumor-infiltrating CD8^+^ T cells in tumor cryosections was conducted. As indicated in Fig. 5f, compared to that in the MC-PLGA@Met-CO_2_ NP-treated and MC-PLGA@siFGL1 NP-treated groups, the percentage of intratumoral CD8^+^ T cells was significantly upregulated relative to the total number of cells in tumors in the MC-PLGA@Met-CO_2_/siFGL1 NP-treated group (*p* < 0.01), which is consistent with the results of the flow cytometry analysis.

### Biosafety assessment and immunohistochemistry analysis

**Fig. 6:**
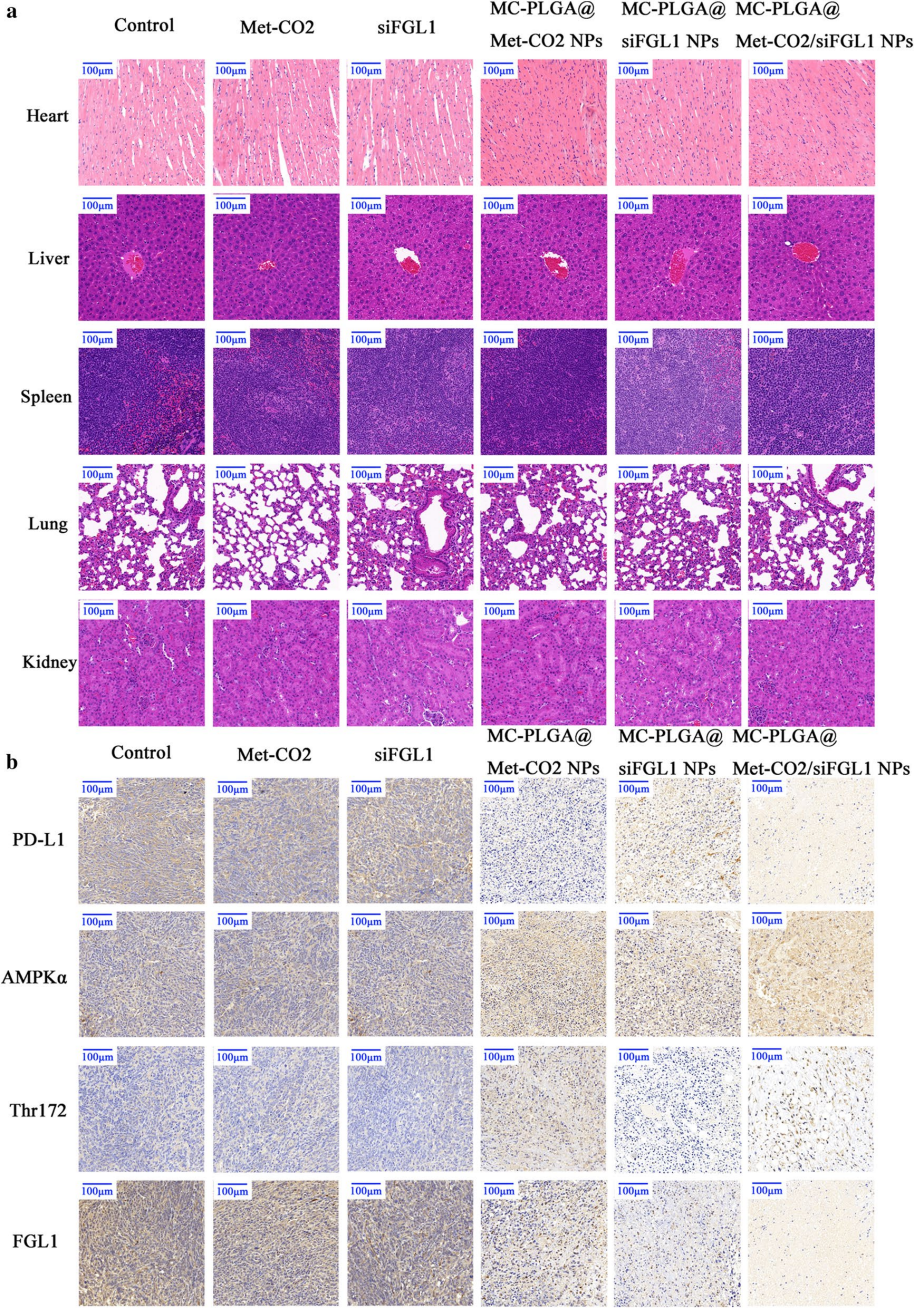
Biosafety estimation of different formulations and immunohistochemistry analysis. **a** Representative histological images of major organs from mice under different treatments. Scale bar, 100 µm. **b** Immunohistochemistry to detect PD-L1, AMPK-α, Thr172, and FGL1 in tumors. The images were acquired with a Leica microscope at low magnification (Scale bar, 100 µm)

The histologic analysis of the main organs (heart, liver, spleen, lung, and kidney) showed no obvious abnormalities in any treatment group, similar to the control group (Fig. 6a). The histology indicates that the systemic toxicity of our therapeutic regimen was low. Furthermore, PD-L1, AMPK-α, Thr172, and FGL1 levels in tumor tissue were evaluated by immunohistochemistry analysis (Fig. 6b). A mass of PD-L1 proteins (brown) appeared in the control, Met-treated, and siFGL1-treated groups, but the MC-PLGA@Met-CO_2_ NP-treated and MC-PLGA@Met-CO_2_/siFGL1 NP-treated groups showed lower PD-L1 levels owing to the reduction of PD- L1 by Met-activated AMPK. AMPK activation was indirectly evaluated by immunohistochemistry analysis of AMPK phosphorylation at Thr172, critical for AMPK activity [48]. As indicated in Fig. 6b, AMPK activation was reflected by increased AMPK Thr172 in the MC-PLGA@Met-CO_2_ NP-treated and MC-PLGA@Met-CO_2_/siFGL1 NP-treated groups compared to that in the other groups. The reduction in FGL1 expression in the MC-PLGA@siFGL1 NP-treated and MC-PLGA@Met-CO_2_/siFGL1 NP-treated groups suppressed the expression of FGL1 in vivo (Fig. 6b).

## Discussion

ICIs have emerged as promising candidates for cancer immunotherapy by reversing the immunosuppressive mechanisms employed by tumors to reinvigorate anti-cancer immunity. In the tumor microenvironment, PD-L1 and its receptor, PD-1, are two typical immune checkpoints. The interaction between PD-1 and PD-L1 can promote tumor immune escape and tumor survival. Thus, blocking the PD-L1/PD-1 axis is considered to be an effective target for cancer immunotherapy. However, the durable response rate following anti-PD-1/PD-L1 immunotherapy remains relatively low in most cases [49, 50]. Some studies have shown that the blocking effect is transient and could be weakened due to active redistribution of intracellular PD-L1 to cell membrane [51, 52]. Thus, maintaining the stability of endogenous PD-L1 is important for PD-L1/PD-1 blockade therapy. Many studies have shown that Met possesses antitumor activity and maintains high cytotoxic T lymphocyte immune surveillance, which may be attributable to Met because it could induce abnormal PD-L1 glycosylation, leading to endoplasmic reticulum-mediated degradation of PD-L1 and thus, facilitate antitumor immunity. In addition, the use of ICIs, which have unique properties with non-overlapping functions, in combination therapy has been a breakthrough in cancer therapeutics [53, 54]. FGL1/LAG3 blockade therapies have proven beneficial in promoting T-cell-mediated immune responses and enhancing antitumor immunity may produce a good synergistic effect on PD-L1/PD-1 blockade therapy. Therefore, we developed a pH-triggered CO_2_ gas-generating hybrid membrane-biomimetic nanoplatform to deliver Met and siFGL1 (MC-PLGA@Met-CO_2_/siFGL1 NPs).

In the present study, the guanidine-modified Met could expand or break open the hybrid membrane-coated nanoparticles in a pH-dependent manner. The membrane coated NPs were designed to improve the condensation and endosomal/lysosomal escape of the encapsulated siRNA. Some reports have suggested that electrostatic interactions could enhance the encapsulation efficiency of gene therapeutic agents in NPs [41, 55–57]. The formation of guanidine-modified Met/siRNA complex used in this study showed a higher amount of siRNA encapsulated in the PLGA NPs compared to that used in other studies [58–60]. Analysis of the size and TEM images of MC-PLGA@Met-CO_2_/siFGL1 NPs showed that under low pH conditions, CO_2_ generated from Met-CO_2_ encapsulated in the PLGA NPs could cause the membrane to expand or break open, resulting in a nanobomb effect. In addition, the drug release results indicated that Met released from MC-PLGA@Met-CO_2_/siFGL1 NPs exhibited a typical sustained and low-pH-triggered release. This pH-responsive release might be attributed to CO_2_ generation in the acidic environment, which could avoid drug leakage during circulation, and induce a faster release with low-pH triggering in the cellular environment. Furthermore, the results of intracellular lysosome escape assay showed that MC-PLGA@Met-CO_2_/FAM-siFGL1 NPs (with CO_2_) exhibit extraordinary endosome escape ability owing to the presence of CO_2_ produced under acidic conditions. This study is consistent with previous reports, in which CO_2_ effectively achieved endosomal escape [40]. In the cell cycle analysis, free Met-CO_2_ showed a slight G1 phase arrest by AMPK activation, which agrees with the results of previous studies [61, 62]. MC-PLGA@Met-CO_2_ NPs triggered the highest level of cell cycle arrest in 4T1 cells, indicating that the delivery of Met-CO_2_, mediated by hybrid membrane-coated NPs, increased the efficacy of cell cycle arrest by Met, which is consistent with the data from the cellular uptake assay. Further, tumor cell killing assay conducted by co-culture has shown an increase in the percentage of apoptosis in Met-CO_2_ and MC-PLGA@Met-CO_2_ NPs-treated groups. This result might be due to the ability of Met-CO_2_ to regulate the immunosuppressive tumor microenvironment by reducing the stability and membrane localization of PD-L1 [16].

FGL1 is a major ligand of LAG3, which diminishes the function of T cells and immune evasion, and is upregulated in several human cancers; its expression is limited in most of the normal tissues and is associated with poor treatment outcomes [18]. Met can block PD-L1-induced inhibitory signals and induce cytotoxic T-lymphocyte activity against cancer cells [16]. To further observe the synergistic effect of Met and siFGL1 when they are co-loaded within hybrid biomimetic system, we collected tumor-infiltrating lymphocytes to analyze the frequency of CD8^+^ T lymphocytes and CD4^+^ T cells and intratumoral cytokine levels at the end of the antitumor treatment in vivo. Our study showed that MC-PLGA@Met-CO2/siFGL1 NPs significantly increased the ratio of CD8^+^ T cells to CD4^+^ T cells and the cytokine levels (*IFN-γ* and *IL-2*). which suggests that the combination therapy can induce an effective antitumor immune response.

In the tumor microenvironment, the hypoxic regions commonly exist in many solid tumors because of hypermetabolism and rapid cell proliferation [63, 64]. Met has been reported to be competent in depressing mitochondrial respiration, resulting in a decrease in endogenous oxygen consumption, thereby combating hypoxia in the tumor environment [65]. In addition, Met can affect the differentiation of TAMs into distinct phenotypes [66, 67]. TAMs display antitumor and pro-tumoral functions during tumor progression, depending on their polarization: M1 (pro-inflammatory) and M2 (anti-inflammatory), respectively. The immunohistochemistry results of hypoxia expression areas suggest that hybrid membrane-coated Met NPs could effectively alleviate tumor hypoxia. Meanwhile, our study indicates that hybrid membrane-coated Met NPs could skew the TAM polarization away from the M2-phenotype to the M1-phenotype, which is known to inhibit tumor progression and angiogenesis. Thus, we have successfully developed hybrid biomimetic camouflaged NPs with multiple targeting capability and demonstrated its efficient co-delivery of Met and siFGL1 to the tumor microenvironment and ameliorated the tumor immunosuppressive microenvironment.

## Conclusions

We developed a pH-responsive hybrid biomimetic membrane-camouflaged PLGA nanoparticle system based on multiple tumor targets and low-pH-activated endosomal/lysosomal escape to co-deliver the FGL1/LAG3 blockade molecule, siFGL1, and immuno-metabolic adjuvant Met. This hybrid biomimetic system, designed for co-delivery of Met and siFGL1, exerted a synergistic immunotherapeutic effect both in vitro and in vivo by blocking the inhibitory signals of PD-L1 and FGL1. Additionally, in vivo antitumor results showed that the Met-loaded hybrid biomimetic membrane-camouflaged PLGA NP delivery system could effectively alleviate tumor hypoxia and induce M1-type differentiation of tumor-related macrophages, thereby improving the tumor immunosuppressive microenvironment. Overall, our results indicated that MC-PLGA@Met-CO_2_/siFGL1 NPs have the potential to become a promising strategy to optimize cancer immunotherapy using combination therapy with multiple ICIs.

## Data Availability

All data generated or analysed during this study are included in this published article.

## Abbreviations

DLS: Dynamic light scattering
FTIR: Fourier-transform infrared
MC: Fused hybrid membrane comprised of macrophage membrane (M) and 4T1 breast cancer cell membrane (C)
Met: Metformin
NP: Nanoparticle
siFGL1: Short interfering RNA targeting fibrinogen-like protein 1 mRNA
NMR: Nuclear magnetic resonance
PLGA: Poly (lactic-*co*-glycolic acid)
TEM: Transmission electron microscope
FAM: Fluorescein amidite
ELISA: Enzyme-linked immunosorbent assay
H&E: Hematoxylin and eosin
IFN-γ: Interferon-γ
IL-2: Interleukin-2
AMPK-α: Adenosine monophosphate-activated protein kinase α
FGL1: Fibrinogen-like protein 1 mRNA
PD-L1: Programmed death-ligand 1

## References

[1] Egen JG, Ouyang W, Wu LC (2020). Human anti-tumor immunity: insights from immunotherapy clinical trials. Immunity.

[2] Esteva FJ, Hubbard-Lucey VM, Tang J, Pusztai L (2019). Immunotherapy and targeted therapy combinations in metastatic breast cancer. Lancet Oncol.

[3] Galon J, Bruni D (2019). Approaches to treat immune hot, altered and cold tumours with combination immunotherapies. Nat Rev Drug Discov.

[4] Kennedy LB, Salama AKS (2020). A review of cancer immunotherapy toxicity. CA Cancer J Clin.

[5] Hegde PS, Chen DS (2020). Top 10 challenges in cancer immunotherapy. Immunity.

[6] Kalbasi A, Ribas A (2020). Tumour-intrinsic resistance to immune checkpoint blockade. Nat Rev Immunol.

[7] Ribas A, Wolchok JD (2018). Cancer immunotherapy using checkpoint blockade. Science.

[8] Sharma P, Allison JP (2015). The future of immune checkpoint therapy. Science.

[9] Vranic S, Cyprian FS, Gatalica Z, Palazzo J (2019). PD-L1 status in breast cancer: current view and perspectives. Semin Cancer Biol.

[10] Yang Z, Wang J, Liu S, Li X, Miao L, Yang B (2020). Defeating relapsed and refractory malignancies through a nano-enabled mitochondria-mediated respiratory inhibition and damage pathway. Biomaterials.

[11] Zuo H, Tao J, Shi H, He J, Zhou Z, Zhang C (2018). Platelet-mimicking nanoparticles co-loaded with W18O49 and metformin alleviate tumor hypoxia for enhanced photodynamic therapy and photothermal therapy. Acta Biomater.

[12] Song X, Feng L, Liang C, Gao M, Song G, Liu Z (2017). Liposomes co-loaded with metformin and chlorin e6 modulate tumor hypoxia during enhanced photodynamic therapy. Nano Res.

[13] Han H, Hou Y, Chen X, Zhang P, Kang M, Jin Q (2020). Metformin-induced stromal depletion to enhance the penetration of gemcitabine-loaded magnetic nanoparticles for pancreatic cancer targeted therapy. J Am Chem Soc.

[14] Xiong Y, Zhao Y, Miao L, Lin CM, Huang L (2016). Co-delivery of polymeric metformin and cisplatin by self-assembled core-membrane nanoparticles to treat non-small cell lung cancer. J Control Release.

[15] Eikawa S, Nishida M, Mizukami S, Yamazaki C, Nakayama E, Udono H (2015). Immune-mediated antitumor effect by type 2 diabetes drug, metformin. Proc Natl Acad Sci USA.

[16] Cha JH, Yang WH, Xia W, Wei Y, Chan LC, Lim SO (2018). Metformin promotes antitumor immunity via endoplasmic-reticulum-associated degradation of PD-L1. Mol Cell.

[17] Andrews LP, Marciscano AE, Drake CG, Vignali DA (2017). LAG3 (CD223) as a cancer immunotherapy target. Immunol Rev.

[18] Wang J, Sanmamed MF, Datar I, Su TT, Ji L, Sun J (2019). Fibrinogen-like protein 1 is a major immune inhibitory ligand of LAG-3. Cell.

[19] Ajina R, Zahavi DJ, Zhang YW, Weiner LM (2019). Overcoming malignant cell-based mechanisms of resistance to immune checkpoint blockade antibodies. Semin Cancer Biol.

[20] George S, Rini BI, Hammers HJ (2019). Emerging role of combination immunotherapy in the first-line treatment of advanced renal cell carcinoma: a review. JAMA Oncol.

[21] Du H, Yi Z, Wang L, Li Z, Niu B, Ren G (2020). The co-expression characteristics of LAG3 and PD-1 on the T cells of patients with breast cancer reveal a new therapeutic strategy. Int Immunopharmacol.

[22] Li G, Gao Y, Gong C, Han Z, Qiang L, Tai Z (2019). Dual- blockade immune checkpoint for breast cancer treatment based on a tumor-penetrating peptide assembling nanoparticle. ACS Appl Mater Interfaces.

[23] Huang LL, Nie W, Zhang J, Xie HY (2020). Cell- membrane- based biomimetic systems with bioorthogonal functionalities. Acc Chem Res.

[24] Li R, He Y, Zhang S, Qin J, Wang J (2018). Cell membrane- based nanoparticles: a new biomimetic platform for tumor diagnosis and treatment. Acta Pharm Sin B.

[25] Liu JM, Zhang DD, Fang GZ, Wang S (2018). Erythrocyte membrane bioinspired near-infrared persistent luminescence nanocarriers for in vivo long- circulating bioimaging and drug delivery. Biomaterials.

[26] Ben-Akiva E, Meyer RA, Yu H, Smith JT, Pardoll DM, Green JJ (2020). Biomimetic anisotropic polymeric nanoparticles coated with red blood cell membranes for enhanced circulation and toxin removal. Sci Adv.

[27] Chen Y, Shen X, Han S, Wang T, Zhao J, He Y (2020). Irradiation pretreatment enhances the therapeutic efficacy of platelet-membrane-camouflaged antitumor nanoparticles. J Nanobiotechnol.

[28] Zhou H, He H, Liang R, Pan H, Chen Z, Deng G (2021). In situ poly I: C released from living cell drug nanocarriers for macrophage-mediated antitumor immunotherapy. Biomaterials.

[29] Shao D, Zhang F, Chen F, Zheng X, Hu H, Yang C (2020). Biomimetic diselenide-bridged mesoporous organosilica nanoparticles as an X-ray-responsive biodegradable carrier for chemo-immunotherapy. Adv Mater.

[30] Liao Y, Zhang Y, Blum NT, Lin J, Huang P (2020). Biomimetic hybrid membrane- based nanoplatforms: synthesis, properties and biomedical applications. Nanoscale Horiz.

[31] Sun W, Deng Y, Zhao M, Jiang Y, Gou J, Wang Y (2021). Targeting therapy for prostate cancer by pharmaceutical and clinical pharmaceutical strategies. J Control Release.

[32] Mai X, Zhang Y, Fan H, Song W, Chang Y, Chen B (2020). Integration of immunogenic activation and immunosuppressive reversion using mitochondrial-respiration-inhibited platelet- mimicking nanoparticles. Biomaterials.

[33] Gong C, Yu X, You B, Wu Y, Wang R, Han L (2020). Macrophage-cancer hybrid membrane-coated nanoparticles for targeting lung metastasis in breast cancer therapy. J Nanobiotechnol.

[34] Faramarzi L, Dadashpour M, Sadeghzadeh H, Mahdavi M, Zarghami N (2019). Enhanced anti- proliferative and pro- apoptotic effects of metformin encapsulated PLGA-PEG nanoparticles on SKOV3 human ovarian carcinoma cells. Artif Cells Nanomed Biotechnol.

[35] Zhao Y, Wang W, Guo S, Wang Y, Miao L, Xiong Y (2016). PolyMetformin combines carrier and anticancer activities for *in vivo* siRNA delivery. Nat Commun.

[36] Mandai M, Hamanishi J, Abiko K, Matsumura N, Baba T, Konishi I (2016). Dual faces of IFNgamma in cancer progression: a role of PD-L1 induction in the determination of pro- and antitumor immunity. Clin Cancer Res.

[37] Kwak JH, Hu JZ, Hoyt DW, Sears JA, Wang C, Rosso KM (2010). Metal carbonation of forsterite in supercritical CO_2_ and H_2_O using solid state ^29^Si, ^13^C NMR spectroscopy. J Phys Chem C.

[38] Tung CH, Wei Z, Leibowitz MJ, Stein S (1992). Design of peptide- acridine mimics of ribonuclease activity. Proc Natl Acad Sci USA.

[39] Wang Y, Ji X, Ruan M, Liu W, Song R, Dai J (2018). Worm- like biomimetic nanoerythrocyte carrying siRNA for melanoma gene therapy. Small.

[40] Xu J, Liu Y, Li Y, Wang H, Stewart S, Van der Jeught K (2019). Precise targeting of POLR2A as a therapeutic strategy for human triple negative breast cancer. Nat Nanotechnol.

[41] Kesharwani P, Gajbhiye V, Jain NK (2012). A review of nanocarriers for the delivery of small interfering RNA. Biomaterials.

[42] Ho W, Zhang XQ, Xu X (2016). Biomaterials in siRNA delivery: a comprehensive review. Adv Healthc Mater.

[43] Hawley SA, Ross FA, Chevtzoff C, Green KA, Evans A, Fogarty S (2010). Use of cells expressing gamma subunit variants to identify diverse mechanisms of AMPK activation. Cell Metab.

[44] Kuhajda FP (2008). AMP- activated protein kinase and human cancer: cancer metabolism revisited. Int J Obes (Lond).

[45] Sherman MA, Suresh MV, Dolgachev VA, McCandless LK, Xue X, Ziru L (2018). Molecular characterization of hypoxic alveolar epithelial cells after lung contusion indicates an important role for HIF-1α. Ann Surg.

[46] Li W, Liu H, Qian W, Cheng L, Yan B, Han L (2018). Hyperglycemia aggravates microenvironment hypoxia and promotes the metastatic ability of pancreatic cancer. Comput Struct Biotechnol J.

[47] Diman NY, Brooks G, Kruithof BP, Elemento O, Seidman JG, Seidman CE (2014). Tbx5 is required for avian and mammalian epicardial formation and coronary vasculogenesis. Circ Res.

[48] Stein SC, Woods A, Jones NA, Davison MD, Carling D (2000). The regulation of AMP-activated protein kinase by phosphorylation. Biochem J.

[49] He C, Duan X, Guo N, Chan C, Poon C, Weichselbaum RR (2016). Core- shell nanoscale coordination polymers combine chemotherapy and photodynamic therapy to potentiate checkpoint blockade cancer immunotherapy. Nat Commun.

[50] Kokolus KM, Haley JS, Koubek EJ, Gowda R, Dinavahi SS, Sharma A (2018). Schweinfurthin natural products induce regression of murine melanoma and pair with anti-PD-1 therapy to facilitate durable tumor immunity. Oncoimmunology.

[51] Wang H, Yao H, Li C, Shi H, Lan J, Li Z (2019). HIP1R targets PD-L1 to lysosomal degradation to alter T cell-mediated cytotoxicity. Nat Chem Biol.

[52] Yao H, Lan J, Li C, Shi H, Brosseau JP, Wang H (2019). Inhibiting PD-L1 palmitoylation enhances T-cell immune responses against tumours. Nat Biomed Eng.

[53] Billan S, Kaidar-Person O, Gil Z (2020). Treatment after progression in the era of immunotherapy. Lancet Oncol.

[54] Gaynor N, Crown J, Collins DM (2020). Immune checkpoint inhibitors: Key trials and an emerging role in breast cancer. Semin Cancer Biol.

[55] Qiu M, Ouyang J, Wei Y, Zhang J, Lan Q, Deng C (2019). Selective cell penetrating peptide- functionalized envelope- type chimeric lipopepsomes boost systemic RNAi therapy for lung tumors. Adv Healthc Mater.

[56] Li Y, Zhang K, Liu P, Chen M, Zhong Y, Ye Q (2019). Encapsulation of plasmid DNA by nanoscale metal-organic frameworks for efficient gene transportation and expression. Adv Mater.

[57] Frede A, Neuhaus B, Klopfleisch R, Walker C, Buer J, Müller W (2016). Colonic gene silencing using siRNA- loaded calcium phosphate/PLGA nanoparticles ameliorates intestinal inflammation in vivo. J Control Release.

[58] Zuo X, Chen Z, Gao W, Zhang Y, Wang J, Wang J (2020). M6A- mediated upregulation of LINC00958 increases lipogenesis and acts as a nanotherapeutic target in hepatocellular carcinoma. J Hematol Oncol.

[59] Byeon Y, Lee JW, Choi WS, Won JE, Kim GH, Kim MG (2018). CD44- targeting PLGA nanoparticles incorporating paclitaxel and FAK siRNA overcome chemoresistance in epithelial ovarian cancer. Cancer Res.

[60] Pina MF, Lau W, Scherer K, Parhizkar M, Edirisinghe M, Craig D (2017). The generation of compartmentalized nanoparticles containing siRNA and cisplatin using a multi-needle electrohydrodynamic strategy. Nanoscale.

[61] Banala VT, Sharma S, Barnwal P, Urandur S, Shukla RP, Ahmad N (2018). Synchronized ratiometric codelivery of metformin and topotecan through engineered nanocarrier facilitates in vivo synergistic precision levels at tumor site. Adv Healthc Mater.

[62] Zhou X, Kuang Y, Liang S, Wang L (2019). Metformin inhibits cell proliferation in SKM-1 cells via AMPK- mediated cell cycle arrest. J Pharmacol Sci.

[63] Patel A, Sant S (2016). Hypoxic tumor microenvironment: opportunities to develop targeted therapies. Biotechnol Adv.

[64] Graham K, Unger E (2018). Overcoming tumor hypoxia as a barrier to radiotherapy, chemotherapy and immunotherapy in cancer treatment. Int J Nanomedicine.

[65] Wheaton WW, Weinberg SE, Hamanaka RB, Soberanes S, Sullivan LB, Anso E (2014). Metformin inhibits mitochondrial complex I of cancer cells to reduce tumorigenesis. Elife.

[66] Wang JC, Sun X (2018). Metformin's antitumour and anti- angiogenic activities are mediated by skewing macrophage polarization. J Cell Mol Med.

[67] Yin X, Han S, Song C, Zou H, Wei Z, Xu W (2019). Metformin enhances gefitinib efficacy by interfering with interactions between tumor- associated macrophages and head and neck squamous cell carcinoma cells. Cell Oncol (Dordr).

